# Supplementary figures and images for: Nucleotide Imbalance, Provoked by Downregulation of Aspartate Transcarbamoylase Impairs Cold Acclimation in Arabidopsis
Source: Molecules. 2023 Feb 7;28(4):1585. doi: 10.3390/molecules28041585 (PMC9959217; doi:10.3390/molecules28041585)

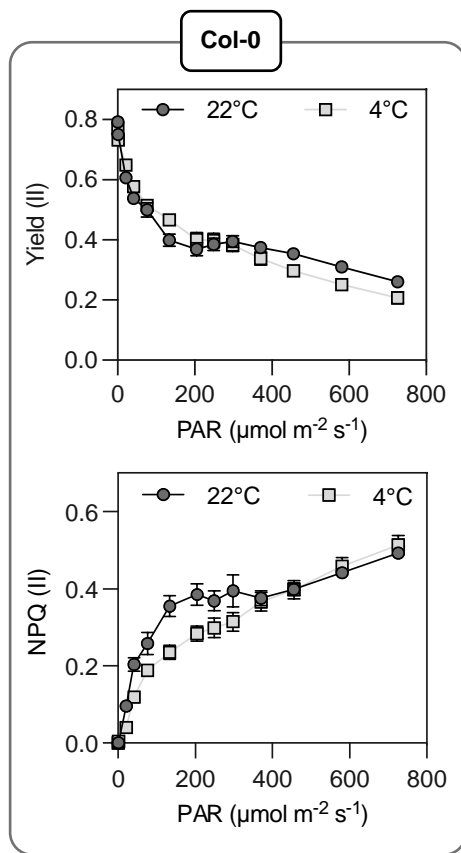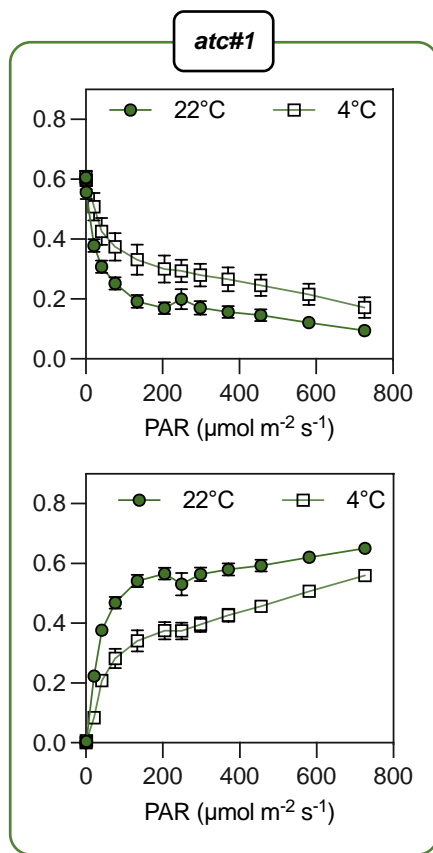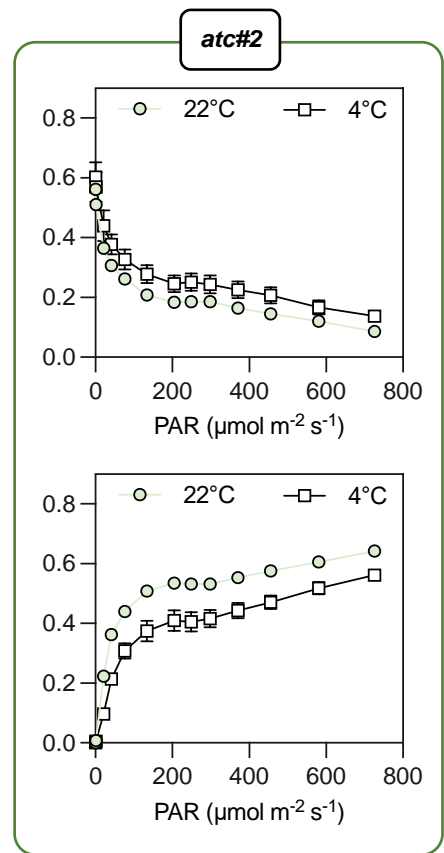

**Figure S1.** Photosynthetic parameters of Col-0 and *ATC* knockdown mutants grown in warm and cold

Supplement: Supplementary file 1 [file molecules-28-01585-s001.zip › Figure S1.pdf]
